# Charge-transfer-activated SERS detection of methylene blue using an ultrastable, reliable, and highly sensitive semiconductor Fe_3_O_4_@C@TiO_2_ nano-platform

**DOI:** 10.1039/d6ra00307a

**Published:** 2026-04-22

**Authors:** Quan-Doan Mai, Dang Thi Hang Trang, Ngo Thi Loan, Ong Van Hoang, Ta Ngoc Bach, Nguyen Quang Hoa, Nhu Hoa Thi Tran, Anh-Tuan Pham, Anh-Tuan Le

**Affiliations:** a Phenikaa University Nano Institute (PHENA), Phenikaa School of Engineering (PSE), Phenikaa University Hanoi 12116 Vietnam doan.maiquan@phenikaa-uni.edu.vn tuan.leanh@phenikaa-uni.edu.vn; b University of Transport Technology Trieu Khuc, Thanh Xuan District Hanoi Vietnam; c Institute of Materials Science (IMS), Vietnam Academy of Science and Technology 18 Hoang Quoc Viet Hanoi 10000 Vietnam; d Faculty of Physics, VNU University of Science, Vietnam National University, Hanoi Thanh Xuan Hanoi Vietnam; e Faculty of Materials Science and Technology, University of Science Ho Chi Minh City Vietnam; f Vietnam National University Ho Chi Minh City Vietnam; g Faculty of Biotechnology, Chemical and Environmental Engineering (BCEE), Phenikaa School of Engineering (PSE), Phenikaa University Hanoi 12116 Vietnam

## Abstract

Surface-enhanced Raman spectroscopy (SERS) is among the most sensitive analytical tools for molecular identification, yet its strong reliance on noble-metal supports (Au, Ag, Cu) still constrains stability, cost, and practical applicability. Recent advances show that certain semiconductors can provide Raman enhancement, offering a metal-free route toward more stable SERS platforms. However, the sensitivity of semiconductor-based substrates remains far inferior to noble-metal systems, with limited enhancement factors (EFs) that restrict real-world applicability. Therefore, it is a formidable yet highly desirable goal to develop an efficient semiconductor SERS substrate with enhancement levels comparable to noble metals. Here, we report Fe_3_O_4_@C@TiO_2_ semiconductor nanostructures as a metal-free SERS substrate capable of activating and amplifying the Raman signal of methylene blue through interfacial states that facilitate efficient semiconductor-molecule charge transfer, resulting in ultrastable, reliable, and highly sensitive sensing performance. Owing to the excellent chemical stability of this platform, the SERS signals of MB remain highly stable over long storage periods, and the substrate exhibits strong reliability with relative standard deviations (RSD) below 5% for both repeatability and reproducibility. Moreover, the presence of transition energy levels within the Fe_3_O_4_@C@TiO_2_ heterostructure substantially enhances the Raman signal, enabling a limit of detection of 76 nM and a maximum EF of 5.4 × 10^5^ – approaching those of noble-metal substrates and significantly higher than most semiconductor-based counterparts. The system further enables accurate MB detection in tap water, yielding recovery values of 93–106%. These results highlight the strong potential of semiconductor heterostructures for stable and practical SERS sensing platforms.

## Abstract 2

We report a metal-free Fe_3_O_4_@C@TiO_2_ heterostructure SERS platform enabling efficient semiconductor-molecule charge transfer *via* interfacial states, achieving sensitivity approaching noble-metal systems with ultrastable and reliable performance.

## Graphical abstract

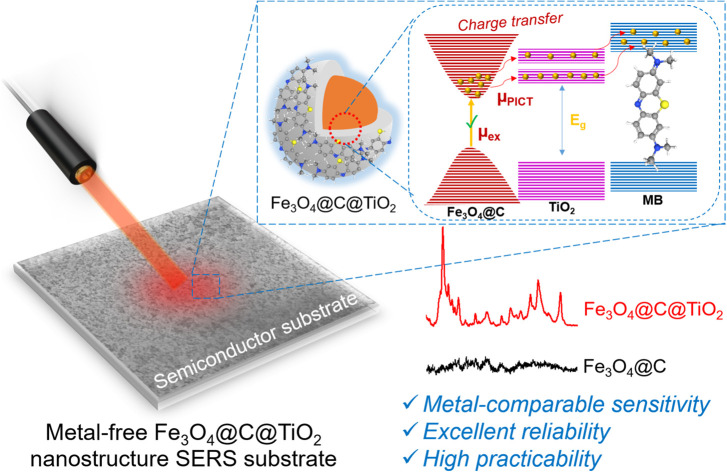

## 1. Introduction

Since its discovery in 1974, surface-enhanced Raman spectroscopy (SERS) has evolved over five decades into a remarkably powerful analytical technique, renowned for its ability to deliver molecular-fingerprint information in a non-destructive manner and, under favorable conditions, to probe down to the single-molecule level.^1–4^ Owing to these unique advantages, SERS has been widely and successfully applied across numerous important fields, including environmental monitoring,^5^ chemical forensics,^6^ food safety,^7^ biomedical diagnostics,^6^ and surface science.^8^ Owing to its far-reaching importance, tremendous experimental and theoretical efforts have been devoted to elucidating the underlying enhancement mechanisms, with the primary goal of designing and developing SERS materials/substrates with higher sensitivity, reliability and practicability for real-life application.^3,9,10^ Although the fundamental origin of SERS enhancement remains under discussion, the phenomenon is generally interpreted through two dominant mechanisms: the electromagnetic (EM) and chemical (CM) mechanisms.^3,11,12^ Noble-metal nanostructures are known to be the most effective SERS substrates due to their ability to support localized surface plasmon resonance (LSPR), which gives rise to the primary EM mechanism.^11^ This mechanism predicts that the local electric field is significantly amplified when excitation occurs within the LSPR region of noble-metal substrates, leading to enhancement factors (EFs) on the order of 10^6^. The second is the CM mechanism, which proposes the formation of charge-transfer complexes between chemisorbed species and the substrate; enhancement is obtained when the excitation frequency resonates with the charge-transfer transition, typically yielding EFs of about 10–100. Because the EM contribution overwhelmingly dominates the overall SERS enhancement, most studies have focused on substrates constructed primarily from noble metals.^13^ However, noble metals present several practical limitations, such as poor chemical and thermal stability, high cost, and challenges associated with fabricating uniformly ordered plasmonic nanostructures.^14–16^ These limitations inherently hinder real-world applications due to reduced stability, insufficient reliability, and the high complexity and cost of fabrication. Consequently, an intrinsic trade-off persists in SERS between high sensitivity and operational reliability. Therefore, the search for alternative materials that can function as SERS substrates while harmonizing sensitivity, reliability, and practicability has become an urgent task.

Recently, several semiconductor materials have been identified as capable of generating measurable SERS signals. Representative examples include ZnO nanosheets,^17^ Cu_2_O nanoparticles,^18^ TiO_2_ nanospheres,^19^ WO_3_ urchin-like structures,^20^ MoO_2_ nanodumbbells,^21^ SnO_2_ nanoparticles,^22^ and InAs/GaAs quantum dots.^23^ Unlike noble-metal substrates, these semiconductor-based systems provide a set of appealing advantages – low production cost, facile synthesis, and, in particular, remarkable chemical durability together with highly reliable Raman responses characterized by excellent repeatability and reproducibility.^22,24,25^ A notable example is the work by Zhang *et al.*, in which MoO_2_ nanodumbbell substrates retained their SERS activity even after being heated at 300 °C for 24 h in air and showed no noticeable degradation under acidic, alkaline, or photochemical conditions. The substrate also produced highly uniform Raman signals, with a relative standard deviation (RSD) as low as 5.2% across multiple measurement spots.^21^ Other reports have further confirmed that fully semiconductor-derived SERS substrates can withstand long-term storage, tolerate harsh chemical environments – including strong acids, strong bases, and extreme temperatures – and still maintain excellent measurement reliability with RSD values below 4%.^24,25^ However, without the EM enhancement, the EFs of these semiconductor substrates are generally only around 10–100, arising primarily from the CM mechanism through charge-transfer complex resonance at the semiconductor-analyte interface under light irradiation.^12^ This presents a major challenge, because without sufficiently high EF values, the resulting SERS signals are weak for trace-level molecular detection, thereby limiting the practical application of semiconductor-based SERS substrates.

Fortunately, several novel strategies have recently been proposed to markedly enhance the SERS performance of semiconductor materials. Since the CM mechanism plays the dominant role in semiconductor-based SERS, these strategies mainly focus on strengthening interfacial interactions between the semiconductor substrate and analyte molecules, thereby facilitating more efficient charge-transfer processes. In 2015, Cong *et al.* reported a metal-comparable enhancement factor of 3.4 × 10^5^ using urchin-like W_18_O_49_ nanowires as a SERS substrate for rhodamine 6G (R6G) by introducing abundant surface oxygen vacancies on W_18_O_49_. The considerable population of oxygen vacancies enriched the surface states of the semiconductor, providing additional intermediate transition levels between W_18_O_49_ and R6G, ultimately enabling a limit of detection (LOD) of 10^−7^ M.^20^ In contrast to the vacancy-engineering approach, Zheng *et al.* (2017) introduced an oxygen-incorporation-assisted strategy for MoS_2_ semiconductor substrate, demonstrating that even trace concentrations of incorporated oxygen could increase the enhancement factor by up to 100 000-fold compared with oxygen-free MoS_2_, while simultaneously enabling a detection limit below 10^−7^ M for R6G analyte.^26^ In 2020, Lin *et al.* proposed a strategy based on constructing crystal-amorphous heterojunctions in core–shell black TiO_2_ nanostructures. The formation of crystal-amorphous interfaces promoted efficient exciton separation, significantly facilitating charge transfer from the crystalline core to the amorphous shell and subsequently to 4-nitrobenzenethiol (4NBT) probe molecules, resulting in a substantial SERS enhancement with an EF of ∼10^5^ and a LOD of 10^−6^ M.^27^ More recently, in 2025, Huu *et al.* developed a MoO_*x*_-overcoated Al-doped ZnO heterostructure as a semiconductor SERS substrate for detecting R6G. The formation of this MoO_*x*_/Al-doped ZnO heterojunction generated interfacial electronic transitions involving MoO_*x*_, Al-doped ZnO, and R6G, which collectively enhanced charge-transfer efficiency and led to improved SERS signals with a LOD as low as 10^−7^ M.^28^ Although these strategies have significantly improved the sensitivity of semiconductor-based SERS substrates and deepened the understanding of their enhancement mechanisms, their detection limits remain largely confined to the ∼10^−7^ M range. This performance remains far inferior to that of noble-metal substrates and still falls short of the requirements for trace-level sensing in practical applications – an area where SERS is expected to excel. Therefore, continued development of semiconductor SERS substrates with further improved sensitivity while preserving their inherent advantages in stability and reliability is still highly desirable.

In this study, we demonstrate Fe_3_O_4_@C@TiO_2_ semiconductor nanostructures as a metal-free SERS substrate capable of achieving a sensitivity level approaching that of noble-metal systems. The heterostructure generates interfacial states that markedly enhance semiconductor-molecule charge transfer, enabling strong activation and amplification of the Raman signal of methylene blue (MB). As a result, the platform delivers highly stable and reliable SERS performance, with MB signals remaining nearly unchanged after prolonged storage and excellent RSDs below 5% for both repeatability and reproducibility. Furthermore, the presence of transition energy levels within the Fe_3_O_4_@C@TiO_2_ architecture significantly boosts the SERS enhancement, yielding a limit of detection of 76 nM and a maximum enhancement factor of 5.4 × 10^5^ – figures that not only surpass most semiconductor-based substrates but also approach those of noble-metal counterparts. The system additionally enables accurate MB quantification in tap water with recovery rates of 93–106%. These findings demonstrate that rationally engineered semiconductor heterostructures can effectively bridge the performance gap with noble metals, offering a promising pathway toward stable, practical, and metal-free SERS sensing.

## 2. Experimental section

### 2.1. Chemicals

Ferrocene (Fe(C_5_H_5_)_2_, 98 wt%), hydrogen peroxide solution (H_2_O_2_, 30 v/v%) were obtained from Sigma-Aldrich (United Kingdom). Titanium tetrachloride (TiCl_4_, ≥99.8 wt%), ammonium hydroxide (NH_4_OH, 28.0–30.0% NH_3_), ethanol (C_2_H_5_OH, 98 v/v%), acetone (C_3_H_6_O, 99 v/v%) and methylene blue (MB, C_16_H_18_ClN_3_S) were supplied by Shanghai Chemical Reagent (China). All reagents were used as received without any additional purification, and double-distilled water was employed for all experimental procedures.

### 2.2. Preparation and characterizations of Fe_3_O_4_@C@TiO_2_ nanomaterials

The Fe_3_O_4_@C@TiO_2_ nanostructures were synthesised through a two-step process, in which Fe_3_O_4_@C was first prepared *via* a hydrothermal method, followed by the *in situ* formation of TiO_2_ nanoparticles using a sol–gel approach. The Fe_3_O_4_@C synthesis procedure was carried out according to our previous report, employing Fe(C_5_H_5_)_2_ as the iron and carbon precursors and H_2_O_2_ as the oxidizing agent.^29^ In a typical synthesis, 0.6 g of Fe(C_5_H_5_)_2_ was first dispersed in 30 mL of acetone and stirred for 15 min. Thereafter, 3 mL of H_2_O_2_ was introduced dropwise, and the mixture was allowed to react under continuous stirring for an additional 30 min. The reaction mixture was subsequently transferred into a Teflon-lined stainless-steel autoclave and heated hydrothermally at 200 °C for 20 h. Once cooled to room temperature, the product was purified by three sequential washes with ethanol and isolated using cold centrifugation. The resulting Fe_3_O_4_@C powder was then dried at 60 °C and stored for later use. To prepare the Fe_3_O_4_@C@TiO_2_ core–shell nanostructures, the TiO_2_ component was formed directly in the presence of Fe_3_O_4_@C using a sol–gel method. The sol–gel procedure employing TiCl_4_ as the titanium precursor and water as the solvent followed our previously published work.^30^ Specifically, Fe_3_O_4_@C was dispersed in 50 mL of distilled water by ultrasonication for 10 minutes, followed by magnetic stirring for 5 minutes. An appropriate amount of TiCl_4_, pre-dispersed in 20 mL of solvent, was then added dropwise into the Fe_3_O_4_@C suspension under continuous stirring. The adsorption interaction between Fe_3_O_4_@C and Ti^4+^ ions was allowed to proceed for 1 hour. Subsequently, 35 mL of C_2_H_5_OH was added to remove the HCl generated from the hydrolysis of TiCl_4_. The pH of the mixture was adjusted to 7 (neutral condition) by the dropwise addition of NH_4_OH. Gel formation and the *in situ* growth of TiO_2_ onto the Fe_3_O_4_@C surface were allowed to occur over 12 hours under static laboratory conditions. The final product was washed thoroughly with distilled water and dried at 60 °C for 4 hours to obtain the Fe_3_O_4_@C@TiO_2_ powder, which was used directly for SERS substrate fabrication.

The morphological features of the Fe_3_O_4_@C and Fe_3_O_4_@C@TiO_2_ nanostructures were characterized using a field-emission scanning electron microscope (FE-SEM, Hitachi S-4800) operated at an accelerating voltage of 5 kV. The elemental composition and spatial distribution of the constituent elements were further examined through energy-dispersive X-ray (EDX) mapping. The crystalline structures of the samples were analyzed by X-ray diffraction (XRD) using a Bruker D5005 diffractometer equipped with Cu Kα radiation (*λ* = 1.5406 Å), operated at 40 kV and 30 mA. Optical properties were assessed using ultraviolet-visible (UV-vis) absorption spectroscopy with a JENWAY 6850 double-beam spectrophotometer and 10 mm quartz cuvettes. Based on the obtained UV-vis absorption spectra, the optical bandgap energies of the Fe_3_O_4_@C and Fe_3_O_4_@C@TiO_2_ semiconductor materials were estimated using Tauc plot analysis. Photoluminescence (PL) spectra were recorded under an excitation wavelength of 380 nm to further probe the electronic structure and interfacial electronic interactions between the TiO_2_ shell and the Fe_3_O_4_@C core in the Fe_3_O_4_@C@TiO_2_ heterostructure.

### 2.3. SERS substrate preparation and measurement

Aluminum (Al) plates were used as the supporting bases for the SERS substrate preparation. Each Al substrate was cut to a size of 1 cm × 1 cm × 0.1 cm and contained a circular active area with a diameter of 0.2 cm. Before deposition, the substrates were rinsed thoroughly with ethanol and air-dried at room temperature (RT). To prepare the SERS-active surfaces, 10 µL of a Fe_3_O_4_@C@TiO_2_ dispersion (1 mg mL^−1^) was drop-cast onto the designated active area and left to dry naturally at RT. This procedure provided a constant material loading of 10 µg per substrate, ensuring uniform coverage. The same preparation protocol was applied consistently for all SERS measurements.

To evaluate the SERS performance of the semiconductor Fe_3_O_4_@C@TiO_2_ substrate, methylene blue (MB) was selected as the probe molecule. A series of aqueous MB solutions with concentrations ranging from 10^−3^ M to 5 × 10^−8^ M, prepared by successive two-fold dilutions, was obtained by dissolving an appropriate amount of MB powder in distilled water. For each SERS measurement, 5 µL of MB solution at the desired concentration was directly drop-cast onto the active area of the Fe_3_O_4_@C@TiO_2_ substrate and allowed to dry at room temperature. The resulting analyte-loaded substrates were then used for SERS signal acquisition using a Raman spectrometer. For real-sample analysis, tap water was collected from a training facility located in Hanoi, Vietnam, and employed directly for SERS experiments without any pretreatment. Raman measurements were carried out using a MacroRaman™ spectrometer (Horiba) equipped with a 785 nm excitation source, as well as an XploRA PLUS Raman microscope (Horiba) using a 532 nm excitation source. To ensure reliable comparison, all measurement parameters were kept identical for both excitation wavelengths. Data collection was conducted through a 100× objective lens with a numerical aperture of 0.90. The laser beam, operated at 45 mW, was delivered onto the sample at an incidence angle of 30°. Under these conditions, the diffraction-limited spot size was estimated to be ∼1.1 µm (based on 1.22*λ*/NA), with an optical penetration depth of approximately 115 nm. The spectrometer is based on a grating-dispersive spectrograph (focal length 115 mm) with a concave aberration-corrected grating, and is equipped with a back-illuminated Syncerity NIR CCD detector (2048 × 70 pixels) thermoelectrically cooled to −50 °C, ensuring high sensitivity and low noise performance. The system provides a spectral resolution of approximately 8 cm^−1^. Each Raman spectrum was recorded with a 30 s integration time and averaged over three sequential acquisitions. Prior to analysis, all spectra were subjected to baseline correction.

## 3. Results and discussion

### 3.1. Characterizations of Fe_3_O_4_@C@TiO_2_ nanostructures

**Fig. 1:**
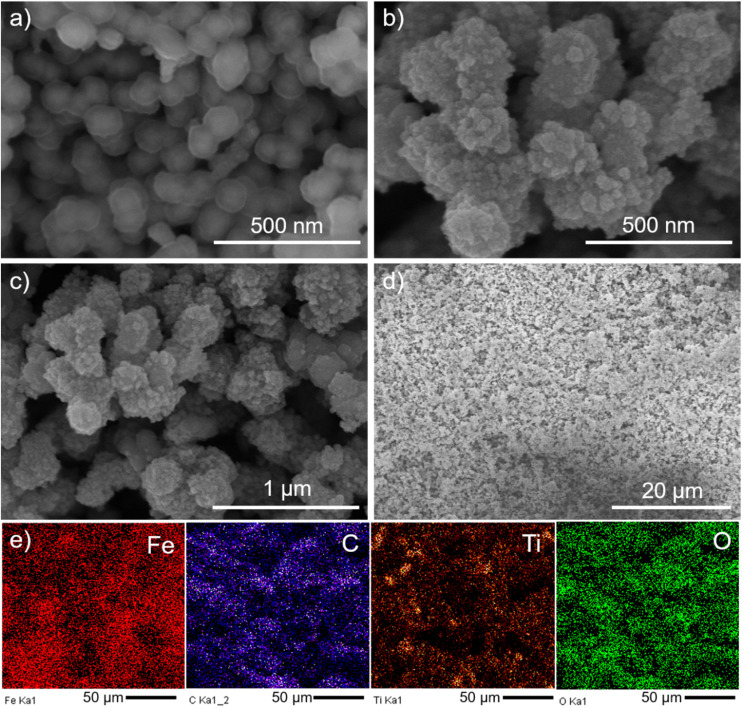
FE-SEM images of Fe_3_O_4_@C (a) and Fe_3_O_4_@C@TiO_2_ nanostructures at different magnifications (b–d); EDX mapping of Fe_3_O_4_@C@TiO_2_ nanostructures (e).

The morphology of Fe_3_O_4_@C and Fe_3_O_4_@C@TiO_2_ nanostructures was examined using FE-SEM, as shown in Fig. 1. The Fe_3_O_4_@C material obtained from the hydrothermal reaction exhibits a spherical shape with uniform size distribution and an average diameter of approximately 130 nm (Fig. 1a). In addition, Fig. 1a clearly reveals a core–shell configuration in which each nanoparticle is surrounded by a shell with lower electron density compared to the core. This low-density shell corresponds to the carbon layer coating the Fe_3_O_4_ core, which possesses a higher electron density. Notably, no significant magnetic-induced aggregation is observed, indicating that the carbon shell effectively prevents the Fe_3_O_4_ magnetic nanoparticles from clustering. This dispersion stability in aqueous media is advantageous for promoting the adsorption of Ti^4+^ ions from the TiCl_4_ precursor during the subsequent sol–gel growth of TiO_2_ on the Fe_3_O_4_@C surface. The carbon shell in the Fe_3_O_4_@C structure therefore plays a dual role: it mitigates magnetic aggregation and simultaneously facilitates the *in situ* formation of TiO_2_. Owing to the excellent adsorption capability of the carbon layer, Fe_3_O_4_@C nanoparticles dispersed in water readily adsorb Ti^4+^ ions within 1 hour of interaction. This is followed by a 12 hour gelation process, during which TiO_2_ forms directly around the carbon shell, yielding the final Fe_3_O_4_@C@TiO_2_ core–shell structure. FE-SEM images of Fe_3_O_4_@C@TiO_2_ at progressively wider views are displayed in Fig. 1b–d. At identical magnification to Fig. 1a and b reveals a pronounced change in both morphology and particle size. While Fe_3_O_4_@C nanoparticles (Fig. 1a) exhibit smaller dimensions and a semi-transparent carbon shell, Fig. 1b shows that the particle diameter increases substantially to approximately 250 nm, and the carbon shell is no longer visible; instead, aggregates of low-transparency nanoparticles surround the surface. This marked morphological evolution during the TiO_2_ formation step is attributed to the direct growth of a TiO_2_ shell onto Fe_3_O_4_@C, leading to increased particle size and modified surface texture. At wider viewing areas (Fig. 1c and d), the Fe_3_O_4_@C@TiO_2_ nanoparticles consistently exhibit enlarged dimensions with uniform growth across individual particles. Fig. 1d further confirms the high degree of uniformity of the Fe_3_O_4_@C@TiO_2_ nanostructures over a large-area region.

The elemental composition and spatial elemental distribution of the Fe_3_O_4_@C@TiO_2_ hybrid nanostructure were further probed by EDX mapping (Fig. 1e). The mapping results clearly identify Fe, C, Ti, and O as the dominant elements, consistent with the expected constituents of Fe_3_O_4_, carbon, and TiO_2_ within the integrated architecture. Notably, all four elements exhibit a highly uniform spatial distribution over the probed area, with no detectable elemental clustering, thereby confirming the absence of phase segregation among the constituent components. The homogeneous distribution of Ti, in particular, provides evidence for the growth of the TiO_2_ shell during the sol–gel deposition process. When considered alongside the FE-SEM observations, the EDX mapping results validate the formation of a heterostructure in which TiO_2_ encapsulates each Fe_3_O_4_@C particle. Such intimate interfacial contact between the TiO_2_ shell and the Fe_3_O_4_@C core is expected to facilitate efficient interfacial electronic coupling, thereby establishing a robust platform for charge-transfer interactions within the Fe_3_O_4_@C@TiO_2_ hybrid – an essential prerequisite for its enhanced SERS activity.

**Fig. 2:**
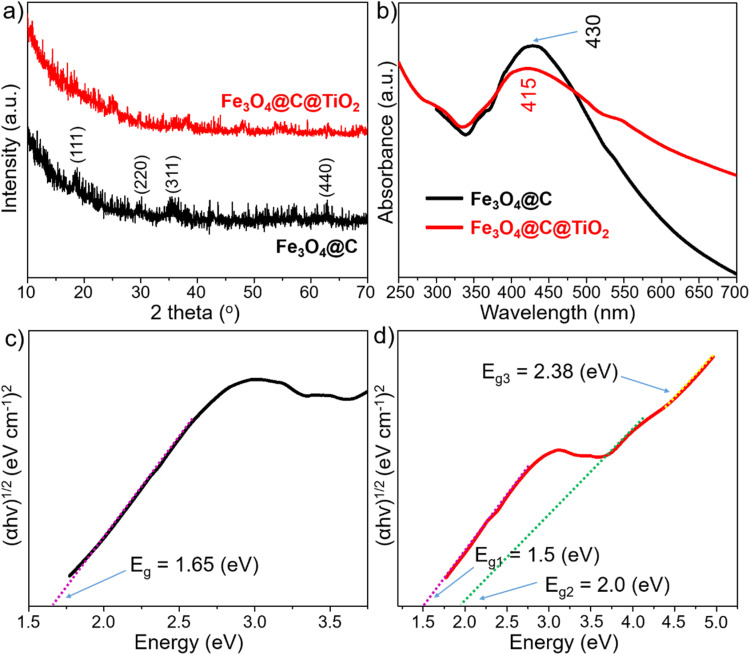
XRD patterns (a) and UV-vis absorption spectra (b) of Fe_3_O_4_@C and Fe_3_O_4_@C@TiO_2_ nanostructure; Tauc plots of Fe_3_O_4_@C (c) and Fe_3_O_4_@C@TiO_2_ (d).

The crystalline properties of the Fe_3_O_4_@C and Fe_3_O_4_@C@TiO_2_ materials were characterized by XRD (Fig. 2a). The XRD pattern of Fe_3_O_4_@C (black curve) displays diffraction peaks at 2*θ* = 18.3°, 30.1°, 35.5°, and 62.6°, which can be assigned to the (111), (220), (311), and (440) lattice planes of inverse-spinel Fe_3_O_4_ with a cubic crystal structure (PDF 01-088-0315). The relatively weak diffraction intensity arising from Fe_3_O_4_ is attributed to the presence of an amorphous carbon shell that partially suppresses the diffraction signal from the core. In the Fe_3_O_4_@C@TiO_2_ system (red curve), these Fe_3_O_4_-related reflections are further attenuated, consistent with the additional deposition of a TiO_2_ shell that further reduces the scattering contribution from the inner Fe_3_O_4_ core. Notably, no characteristic reflections corresponding to crystalline TiO_2_ are observed, indicating that the TiO_2_ shell formed *via* the sol–gel process at room temperature is predominantly amorphous. The significant suppression of Fe_3_O_4_ diffraction in the Fe_3_O_4_@C@TiO_2_ pattern suggests intimate interfacial contact between the TiO_2_ shell and the Fe_3_O_4_@C core. This structural interpretation is strongly supported by the UV-vis absorption spectra (Fig. 2b), in which the Fe_3_O_4_@C@TiO_2_ sample exhibits a pronounced absorption shoulder near ∼415 nm along with an extended visible-light absorption tail – features that are absent in Fe_3_O_4_@C. Whereas Fe_3_O_4_@C shows a dominant absorption maximum at ∼430 nm, the incorporation of TiO_2_ induces a blue-shifted absorption onset and broadens the absorption into the 500–700 nm region. These spectral changes reflect the creation of interfacial electronic interactions between Fe_3_O_4_@C and TiO_2_, giving rise to interfacial electronic states within the hybrid nanostructure. This optical restructuring is further confirmed by Tauc plot analysis.^31^ Fe_3_O_4_@C exhibits a single linear region corresponding to a bandgap of 1.65 eV (Fig. 2c), whereas Fe_3_O_4_@C@TiO_2_ displays three distinct linear regimes (Fig. 2d), yielding optical transition energies of 1.50 eV, 2.00 eV, and 2.38 eV. The lowest-energy transition at 1.50 eV, which is smaller than the intrinsic bandgap of Fe_3_O_4_@C, indicates the formation of interfacial mid-gap states and charge-transfer pathways at the Fe_3_O_4_@C–TiO_2_ junction, effectively narrowing the apparent bandgap of the Fe_3_O_4_@C core. The two higher-energy transitions at 2.00 eV and 2.38 eV are attributed to optical absorption features originating from the amorphous TiO_2_ shell. The emergence of multiple absorption regimes demonstrates that the TiO_2_ coating not only introduces an additional semiconductor phase but also fundamentally reorganizes the electronic landscape across the Fe_3_O_4_@C@TiO_2_ heterostructure. These structural and optical characteristics provide compelling evidence for the formation of a well-defined heterojunction between Fe_3_O_4_@C and TiO_2_. Such a heterojunction is expected to facilitate efficient interfacial charge transfer, which is highly advantageous for semiconductor-based SERS platforms operating through the CM mechanism.

**Fig. 3:**
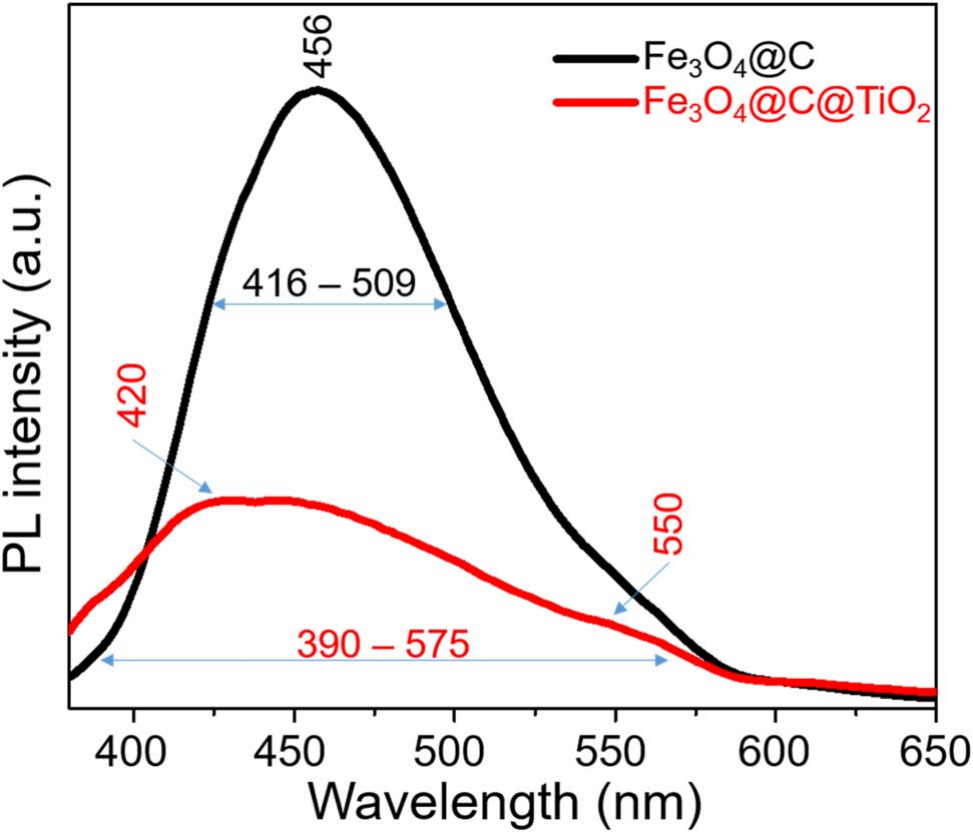
PL spectra of Fe_3_O_4_@C and Fe_3_O_4_@C@TiO_2_ nanostructures.

PL measurements were performed to further probe the electronic structure of the Fe_3_O_4_@C and Fe_3_O_4_@C@TiO_2_ systems and the interfacial electronic interactions between the Fe_3_O_4_@ core and the TiO_2_ shell (Fig. 3). The Fe_3_O_4_@C sample exhibits a strong and broad emission band centered at ∼456 nm, which is attributed to radiative recombination through surface and defect-related states. In contrast, the Fe_3_O_4_@C@TiO_2_ heterostructure shows a markedly quenched PL intensity over the entire spectral range, accompanied by a noticeable spectral broadening (390–575 nm). The pronounced suppression of PL intensity indicates that radiative recombination is effectively inhibited in the heterostructure, suggesting the emergence of efficient non-radiative pathways associated with charge carrier migration across the Fe_3_O_4_@C/TiO_2_ interface. The broadened emission profile further reflects the involvement of multiple electronic states, consistent with the formation of additional interfacial states in the hybrid system. This behavior correlates well with the UV-vis and Tauc results, where the Fe_3_O_4_@C@TiO_2_ sample exhibits an extended absorption tail and multiple optical transition energies (1.50, 2.00, and 2.38 eV), indicating the presence of mid-gap and interfacial electronic states. These states provide energetically accessible pathways for carrier transfer, enabling charge delocalization across the interface and suppressing radiative recombination, as evidenced by the PL quenching. Taken together, the PL, UV-vis, and Tauc analyses consistently demonstrate that the TiO_2_ coating induces significant electronic structure reorganization and establishes a well-defined Fe_3_O_4_@C/TiO_2_ heterojunction. The resulting energy-level alignment and interfacial electronic states facilitate efficient charge-transfer pathways, which are essential for activating the charge-transfer (CM)-driven SERS enhancement in the Fe_3_O_4_@C@TiO_2_ system.

### 3.2. SERS sensing sensitivity and mechanism of Fe_3_O_4_@C@TiO_2_ nanostructures

**Fig. 4:**
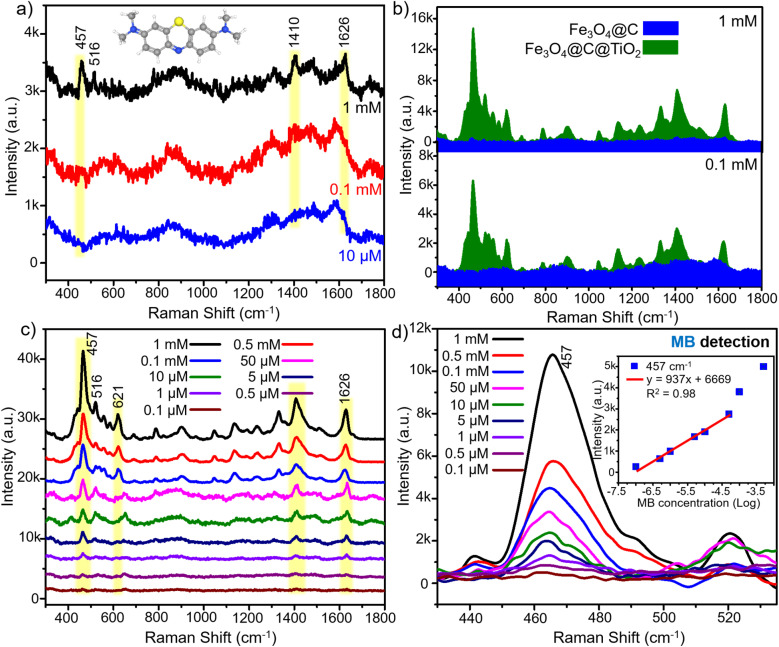
SERS spectra of MB at high concentration obtained on the Fe_3_O_4_@C substrate (a); comparison of SERS intensities of MB collected on Fe_3_O_4_@C@TiO_2_ and Fe_3_O_4_@C substrates (b); SERS spectra of MB over the concentration range from 1 mM to 0.1 µM on the Fe_3_O_4_@C@TiO_2_ substrate (c); and SERS intensity at the characteristic 457 cm^−1^ peak of MB recorded on the Fe_3_O_4_@C@TiO_2_ substrate along with the corresponding linear relationship between MB concentration and SERS intensity (d).

Strategies based on constructing heterojunctions within hybrid semiconductor materials have been widely reported to enhance SERS performance. To elucidate the beneficial role of the heterointerfaces formed in the Fe_3_O_4_@C@TiO_2_ architecture, a comparative SERS investigation was conducted using Fe_3_O_4_@C and Fe_3_O_4_@C@TiO_2_ as the sensing substrates. Methylene blue (MB) – a widely used organic dye known for its persistence in aqueous environments and associated environmental and health concerns – was selected as the probe molecule. We first evaluated the SERS performance of the Fe_3_O_4_@C substrate (details of the substrate preparation are presented in Section 2.3), and the results are presented in Fig. 4a. At a high MB concentration of 1 mM, several characteristic Raman bands are detectable at 457, 516, 1410, and 1626 cm^−1^. These peaks correspond to the C–N–C skeletal deformation mode (457 cm^−1^ and 516 cm^−1^), the C–N/C–C stretching vibrations of the aromatic skeleton (1410 cm^−1^), and the aromatic C=C stretching mode (1626 cm^−1^), respectively.^32,33^ This indicates that Fe_3_O_4_@C is capable of detecting MB at relatively high concentrations. However, the intensities of these Raman features remain weak. More importantly, upon decreasing the MB concentration to 0.1 mM and 10 µM, all characteristic MB peaks disappear, demonstrating the limited SERS sensitivity of Fe_3_O_4_@C. Thus, although the semiconductor-based Fe_3_O_4_@C substrate can detect MB, its SERS performance is restricted to concentrations as high as 1 mM, making it unsuitable for practical analytical applications and far inferior to the capabilities typically achieved using noble-metal-based SERS substrates.

**Fig. 5:**
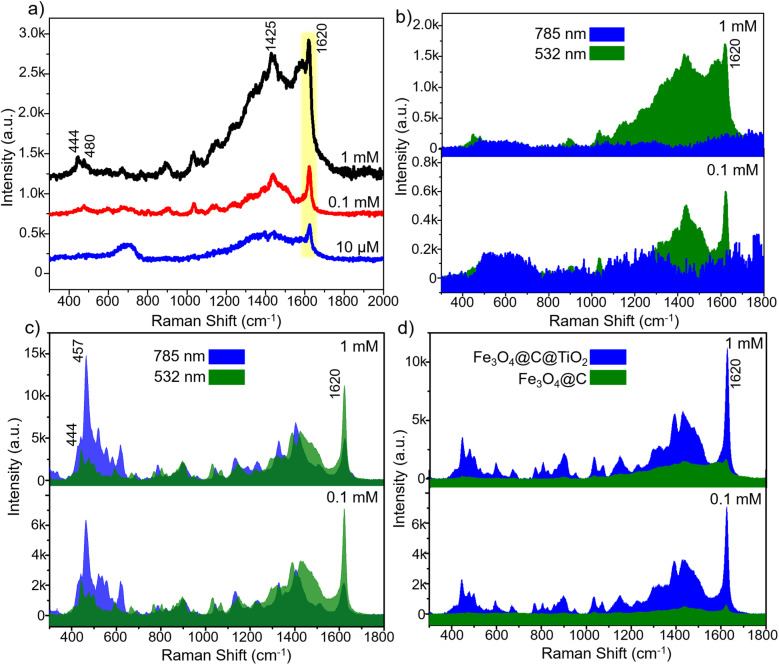
SERS spectra of MB obtained on the Fe_3_O_4_@C substrate under 532 nm excitation (a); comparison of SERS spectra of MB on Fe_3_O_4_@C under 785 nm and 532 nm excitation (b); comparison of SERS spectra of MB on Fe_3_O_4_@C@TiO_2_ under 785 nm and 532 nm excitation (c) and comparison of SERS spectra of MB on Fe_3_O_4_@C and Fe_3_O_4_@C@TiO_2_ under 532 nm excitation (d).

To further examine whether the limited SERS performance of Fe_3_O_4_@C originates from insufficient excitation energy rather than intrinsic inactivity, additional SERS measurements were performed using a higher-energy excitation source (532 nm), as shown in Fig. 5. Fig. 5a presents the SERS spectra of MB collected on the Fe_3_O_4_@C substrate at concentrations of 1 mM, 0.1 mM, and 10 µM under 532 nm excitation, with all experimental parameters kept identical to those used for the 785 nm measurements (Section 2.3). Under these conditions, characteristic Raman peaks of MB are clearly observed, with enhanced intensity compared to those obtained under 785 nm excitation. A comparison between the two excitation wavelengths (Fig. 5b) reveals notable differences in both spectral intensity and peak dominance. Under 785 nm excitation, the SERS response is dominated by the low-frequency vibrational mode at 457 cm^−1^, corresponding to the C–N–C skeletal deformation. In contrast, under 532 nm excitation, the aromatic C=C stretching mode at 1620 cm^−1^ becomes the most strongly enhanced feature. Importantly, the persistence of the 1620 cm^−1^ peak even at 10 µM further demonstrates that the Fe_3_O_4_@C substrate can be effectively activated under higher-energy excitation. These results clearly indicate that the weak SERS performance observed under 785 nm does not arise from intrinsic inactivity of the Fe_3_O_4_@C substrate, but rather from insufficient excitation energy to effectively trigger charge-transfer and resonance processes.

The SERS sensing capability of the Fe_3_O_4_@C@TiO_2_ nanostructured substrate was first evaluated at relatively high MB concentrations (1 mM and 0.1 mM) in comparison with Fe_3_O_4_@C under 785 nm excitation, and the results are presented in Fig. 4b. Remarkably, the SERS signals produced by Fe_3_O_4_@C@TiO_2_ are significantly stronger than those obtained from Fe_3_O_4_@C. At 1 mM MB, the SERS spectrum collected on Fe_3_O_4_@C@TiO_2_ exhibits well-defined characteristic bands of MB, with the intensity at 457 cm^−1^ being enhanced by a factor of approximately 32 relative to Fe_3_O_4_@C. Similar improvements are observed for the other characteristic Raman modes. Importantly, at 0.1 mM MB – where Fe_3_O_4_@C completely fails to produce detectable Raman signatures – the Fe_3_O_4_@C@TiO_2_ substrate still delivers sharp and intense MB peaks. To further examine the excitation-dependent behavior, the SERS response of MB on Fe_3_O_4_@C@TiO_2_ at concentrations of 1 mM and 0.1 mM was also investigated under 532 nm excitation, as shown in Fig. 5c. Notably, the SERS intensity obtained on Fe_3_O_4_@C@TiO_2_ exhibits no significant difference between 785 nm and 532 nm excitation. This behavior is in clear contrast to the Fe_3_O_4_@C substrate, which shows strong SERS activity under 532 nm but remains weak under 785 nm excitation. These results indicate that, unlike Fe_3_O_4_@C, the Fe_3_O_4_@C@TiO_2_ system is not limited by excitation energy and can effectively support charge-transfer processes across a broader excitation window. Furthermore, under identical 532 nm excitation conditions – where the SERS activity of Fe_3_O_4_@C is already activated – the Fe_3_O_4_@C@TiO_2_ substrate still exhibits significantly higher Raman intensity than Fe_3_O_4_@C, as shown in Fig. 5d. This observation demonstrates that the superior SERS performance of Fe_3_O_4_@C@TiO_2_ does not originate solely from excitation-induced activation, but rather from its intrinsically optimized electronic structure. The presence of interfacial states and efficient charge-transfer pathways in the heterostructure enables stronger and more robust SERS enhancement compared to the Fe_3_O_4_@C substrate, even when both systems are activated under high-energy excitation.

Further measurements carried out across a broader concentration range – from 1 mM down to 10 nM using two-fold dilution steps – are shown in Fig. 4c. A systematic decrease in Raman intensity is observed as the MB concentration decreases. Even at 1 µM, all characteristic MB peaks remain clearly distinguishable. Notably, at an extremely low concentration of 0.1 µM (100 nM), the diagnostic MB modes at 457, 1410, and 1626 cm^−1^ are still detectable and remain distinguishable from the background noise, demonstrating the outstanding SERS sensitivity of the Fe_3_O_4_@C@TiO_2_ semiconductor substrate. For quantitative analysis, the 457 cm^−1^ band was selected to evaluate the correlation between MB concentration and SERS intensity (Fig. 4d). A highly linear relationship is obtained over the range from 50 µM to 50 nM, with a correlation coefficient of *R*^2^ = 0.98 and a fitted regression equation of *y* = 937 × *x* + 6669 (where *x* is the logarithm of MB concentration and *y* is the corresponding SERS intensity). Based on this linear calibration and the LOD calculation method described in the SI, the detection limit for MB using the Fe_3_O_4_@C@TiO_2_ SERS substrate is determined to be 76 nM. In addition, the maximum enhancement factor (EF), calculated using the method detailed in the SI, reaches 5.4 × 10^5^. A comparative evaluation of the SERS performance of Fe_3_O_4_@C@TiO_2_ with previously reported semiconductor-based a nd noble-metal-based substrates is summarized in Table 1. The Fe_3_O_4_@C@TiO_2_ substrate exhibits significantly superior sensitivity compared to typical semiconductor SERS substrates, which generally achieve detection limits in the range of 1 to 0.1 µM. This range is consistent with semiconductor substrates employing different probe molecules (*e.g.*, 4-aminothiophenol, rhodamine 6G, and 4-nitrobenzenethiol). In addition, for semiconductor SERS substrates specifically used for MB detection, such as ZnO microtubes and Na_*y*_WO_3−*x*_ nanosheets, the reported detection limits are around 0.1 µM. Notably, both the EF and LOD achieved by Fe_3_O_4_@C@TiO_2_ approach those of noble-metal SERS platforms such as Ag/rGO and Fe_3_O_4_/GO/Ag, which are well known for their strong enhancement capabilities. These results highlight the exceptional SERS performance of the Fe_3_O_4_@C@TiO_2_ semiconductor substrate, surpassing conventional semiconductor-based systems and exhibiting sensitivity approaching that of noble-metal SERS substrates.

**Table 1:** Comparison of the SERS sensing sensitivity of the Fe_3_O_4_@C@TiO_2_ semiconductor substrate with other semiconductor-based SERS substrates and noble-metal SERS substrates for MB detection, in terms of enhancement factor (EF) and limit of detection (LOD)

SERS substrate	Type	Analyte	EF	LOD (M)	Ref.
Double-shelled ZnO hollow microspheres	Semiconductor	4-Aminothiophenol	1.2 × 10^4^	1 × 10^−7^	34
Urchin-like W_18_O_49_ nanowires	Semiconductor	Rhodamine 6G	3.4 × 10^5^	1 × 10^−7^	20
Core-shell black TiO_2_ nanostructures	Semiconductor	4-Nitrobenzenethiol	∼10^5^	1 × 10^−6^	27
MoO_*x*_/Al-doped ZnO heterostructures	Semiconductor	Rhodamine 6G	—	1 × 10^−7^	28
ZnO microtube	Semiconductor	Methylene blue	6.1 × 10^5^	1 × 10^−7^	35
Na_*y*_WO_3−*x*_ nanosheet	Semiconductor	Methylene blue	—	1 × 10^−7^	36
Fe_3_O_4_/GO/Ag composite microspheres	Noble metal	Methylene blue	—	1 × 10^−9^	37
Ag/rGO nanocomposite	Noble metal	Methylene blue	5.33 × 10^5^	1 × 10^−9^	38
Fe_3_O_4_@C@TiO_2_ nanostructures	Semiconductor	Methylene blue	5.4 × 10^5^	76 × 10^−9^	This work

**Fig. 6:**
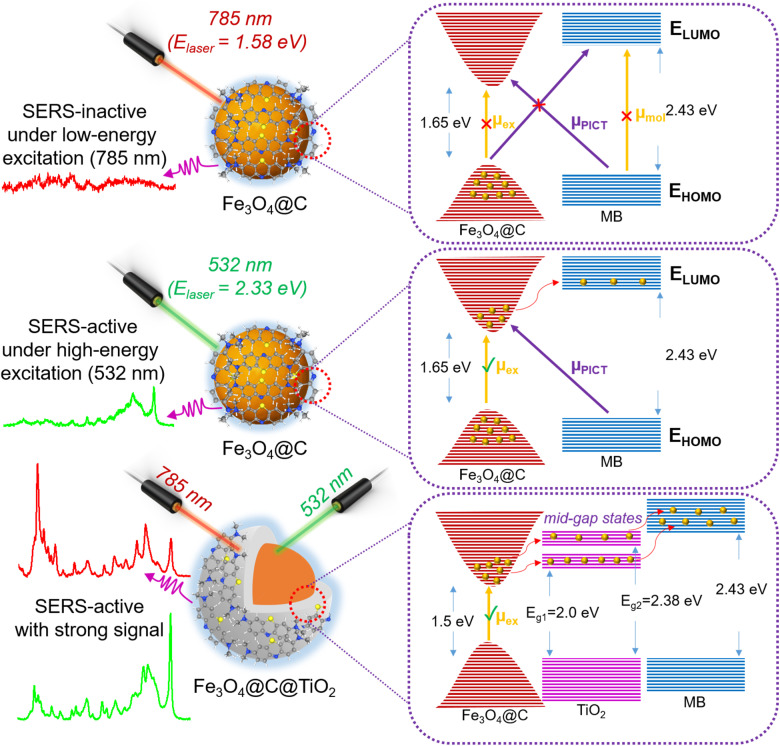
Schematic illustration of the interfacial electronic mechanism responsible for the markedly enhanced SERS activity of the Fe_3_O_4_@C@TiO_2_ nanostructure compared with Fe_3_O_4_@C toward MB molecules under different excitation regimes.

The markedly enhanced SERS response of MB on the Fe_3_O_4_@C@TiO_2_ substrate, compared with Fe_3_O_4_@C, provides valuable insights into the underlying semiconductor-based SERS mechanisms. Fig. 6 illustrates the proposed mechanism, which centers on the substantial improvement of charge-transfer (CT) efficiency between the semiconductor substrate and the analyte molecules, enabled by the formation of heterojunctions within the Fe_3_O_4_@C@TiO_2_ architecture. Taking inspiration from the pioneering theoretical framework of Lombardi *et al.* for semiconductor SERS, we consider the possible involvement of three resonance pathways – exciton resonance (µ_ex_), photoinduced charge-transfer resonance (µ_PICT_), and molecular resonance (µ_mol_).^39^ These resonances correspond to distinct photoexcitation processes capable of enhancing Raman scattering by increasing the polarizability of the coupled molecule-semiconductor system. Exciton resonance (µ_ex_) originates from the generation of electron–hole pairs within the semiconductor, thereby amplifying the surface electronic susceptibility. Photoinduced charge-transfer resonance (µ_PICT_) arises when incident photons promote electrons between the semiconductor band edges and the molecular orbitals of adsorbed MB, creating a vibronically coupled charge redistribution that strongly boosts Raman signals. Molecular resonance (µ_mol_) refers to the intrinsic resonance Raman process when the excitation energy matches the electronic absorption of the analyte. When any of these resonances is brought into energetic alignment with the excitation laser, the Raman polarizability derivative – dictating Raman intensity – is substantially increased, leading to pronounced SERS amplification. Beyond these resonance considerations, recent theoretical models from our group and others have demonstrated that a favorable energetic offset between the semiconductor band edges (valence band and conduction band) and the highest occupied molecular orbitals (HOMO) and lowest unoccupied molecular orbitals (LUMO) levels of the analyte can significantly promote SERS enhancement by facilitating efficient charge transfer.^40–42^

In the case of the Fe_3_O_4_@C substrate, where only weak SERS enhancement is observed for MB, the intrinsic bandgap of 1.65 eV exceeds the excitation energy of the 785 nm laser (1.58 eV). Thus, exciton resonance (µ_ex_) cannot be activated (Fig. 6). The HOMO–LUMO gap of MB (2.43 eV) is also much larger than the photon energy, ruling out the possibility of µ_mol_ activation. Furthermore, the large energetic mismatch between the band edges of Fe_3_O_4_@C and the MB molecular orbitals leaves no viable CT transition pathway, effectively suppressing µ_PICT_. Consequently, Fe_3_O_4_@C yields only minimal SERS enhancement. This interpretation is further corroborated by the excitation-dependent SERS results discussed above (Fig. 5). Under higher-energy excitation (532 nm, 2.33 eV), the photon energy becomes sufficient to overcome the energetic mismatch in the Fe_3_O_4_@C system, enabling exciton generation (µ_ex_) and partially activating charge-transfer processes (Fig. 6). As a result, detectable SERS signals are observed for Fe_3_O_4_@C under 532 nm excitation. However, the enhancement remains limited, indicating that such activation is primarily excitation-driven rather than arising from an intrinsically favorable electronic structure. In contrast, the Fe_3_O_4_@C@TiO_2_ heterostructure exhibits strong SERS activity under both low-energy (785 nm) and high-energy (532 nm) excitation conditions, as illustrated in Fig. 5c and summarized schematically in Fig. 6. Notably, the SERS intensity of Fe_3_O_4_@C@TiO_2_ shows only minor variation between the two excitation wavelengths, demonstrating that the system is not restricted by excitation energy. This behavior highlights the critical role of interfacial electronic structure in governing SERS performance. To understand the origin of this excitation-independent behavior, it is necessary to consider the electronic structure modification induced by TiO_2_ coating. In this regard, coating Fe_3_O_4_@C with TiO_2_ produces a heterostructure that fundamentally reorganizes the interfacial electronic landscape. The Fe_3_O_4_@C@TiO_2_ interface undergoes band alignment modification, the introduction of mid-gap states, and an increase in surface state density – all of which open new, energetically favorable CT pathways. UV-vis and Tauc analyses (Fig. 2b–d) reveal a reduced apparent bandgap of ∼1.50 eV for Fe_3_O_4_@C@TiO_2_, the emergence of a prominent shoulder at ∼415 nm, and multiple linear regimes in the Tauc plot, all indicative of mid-gap and CT-related transitions. The band bending formed at the Fe_3_O_4_@C@TiO_2_ interface further promotes charge separation and transfer. This electronic restructuring is further supported by PL measurements, where the Fe_3_O_4_@C@TiO_2_ heterostructure exhibits a markedly quenched and broadened emission compared to Fe_3_O_4_@C, indicating suppressed radiative recombination and the presence of efficient non-radiative pathways associated with interfacial charge transfer. With a bandgap lowered to 1.50 eV, the 785 nm excitation becomes sufficient to trigger exciton generation (µ_ex_) in Fe_3_O_4_@C@TiO_2_, injecting electrons into the conduction band. The presence of intermediate energy states – corresponding to transition energies of 2.00 eV and 2.38 eV – creates additional “stepping-stone” levels, facilitating electron migration toward the LUMO of MB through an energetically favorable bridging mechanism, thereby activating a strong µ_PICT_ process. Furthermore, under high-energy excitation (532 nm), both Fe_3_O_4_@C and Fe_3_O_4_@C@TiO_2_ are capable of generating excitons and supporting charge-transfer transitions. Nevertheless, as demonstrated experimentally (Fig. 5d), the Fe_3_O_4_@C@TiO_2_ heterostructure consistently delivers significantly higher Raman intensity than Fe_3_O_4_@C under identical conditions. This clearly indicates that the superior SERS performance of Fe_3_O_4_@C@TiO_2_ cannot be attributed solely to excitation-induced activation, but instead arises from the presence of interfacial states and multi-step charge-transfer pathways that enable more efficient electronic coupling with the analyte. The mid-gap states introduced at the Fe_3_O_4_@C@TiO_2_ interface act as intermediate energy levels, facilitating stepwise charge transfer toward the LUMO of MB. This “stepping-stone” mechanism enhances the probability of µ_PICT_ transitions and strengthens the overall charge-transfer resonance. As a result, even when both systems are activated under high-energy excitation, the heterostructure maintains a clear advantage in SERS enhancement. Importantly, this demonstrates that rational electronic structure engineering *via* semiconductor heterostructure formation enables efficient SERS activation under low-energy NIR excitation (785 nm), which is more advantageous for practical applications due to reduced photodegradation and suppressed fluorescence background. Thus, the heterojunction between Fe_3_O_4_@C and TiO_2_, together with the formation of intermediate electronic states, simultaneously enables both µ_ex_ and µ_PICT_ in the Fe_3_O_4_@C@TiO_2_/MB system. This dual activation may lead directly to the exceptionally strong experimental SERS enhancement – EF up to 5.4 × 10^5^ and LOD down to 76 nM. Taken together, the outstanding SERS performance of Fe_3_O_4_@C@TiO_2_ can be understood through a unified mechanism: (i) heterojunction formation introduces additional energy levels that align charge-transfer transitions with the excitation photon energy, and (ii) enhanced exciton resonance strengthens intensity borrowing toward the CT resonance, amplifying the overall Raman response. Therefore, engineering heterojunctions within semiconductor architectures provides a powerful route for tuning substrate energy levels and optimizing both charge-transfer and excitonic transitions – key prerequisites for achieving strong semiconductor-based SERS enhancement for specific target molecules.

### 3.3. SERS sensing reliability of semiconductor Fe_3_O_4_@C@TiO_2_ nanostructures

**Fig. 7:**
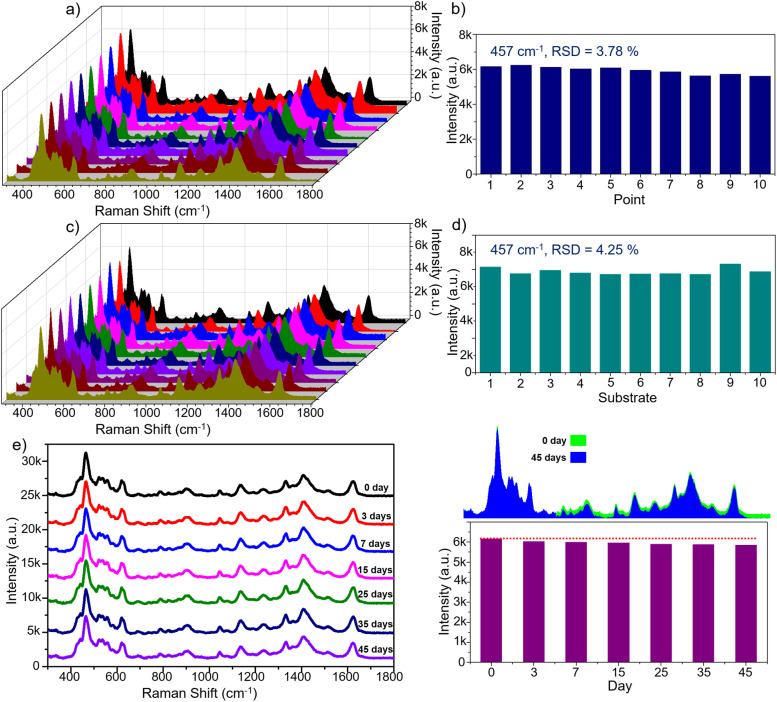
Evaluation of the repeatability of the Fe_3_O_4_@C@TiO_2_ SERS substrate by collecting SERS spectra from 10 randomly selected spots on a single substrate (a) and calculating the RSD based on the intensity of the 457 cm^−1^ peak (b); assessment of reproducibility by acquiring SERS signals from 10 independently fabricated substrates and determining the corresponding RSD using the 457 cm^−1^ peak intensity (c and d); and analysis of the signal stability of the Fe_3_O_4_@C@TiO_2_ substrate through monitoring SERS responses over different storage periods (e).

Having demonstrated a near metal-comparable level of sensitivity for the metal-free Fe_3_O_4_@C@TiO_2_ substrates, it is essential to further evaluate the reliability of their SERS response, which determines whether the substrate can deliver stable and reproducible signals for practical sensing applications. Semiconductor-based SERS substrates, governed predominantly by the CM enhancement mechanism, generally offer higher stability and signal uniformity compared with noble-metal substrates, whose EM enhancement critically depends on the spatial distribution and uniformity of plasmonic “hot spots”, which are inherently difficult to control. The reliability of the Fe_3_O_4_@C@TiO_2_ substrate was evaluated using three key metrics: repeatability, reproducibility, and stability, as summarized in Fig. 7. Fig. 7a presents the repeatability assessment, in which ten SERS spectra were collected from ten randomly selected positions on a single substrate using MB at 0.1 mM. All characteristic MB peaks appear clearly and consistently across the spectra. Quantitatively, the 457 cm^−1^ band was used to determine the intensity variation and calculate the relative standard deviation (RSD) (Fig. 7b), yielding an RSD of 3.78%. The reproducibility of the substrate was further examined by fabricating ten independent Fe_3_O_4_@C@TiO_2_ substrates and recording SERS spectra of 0.1 mM MB from each of them. As shown in Fig. 7c and d, all ten spectra display highly consistent peak positions and intensities, resulting in an RSD of 4.25%. Both RSD values – 3.78% for repeatability and 4.25% for reproducibility – are below 5%, demonstrating that the Fe_3_O_4_@C@TiO_2_ semiconductor substrate delivers exceptional signal reliability. This high level of uniformity and reproducibility is critical for real-world analytical applications, ensuring that SERS signals remain consistent across different measurement locations and across multiple substrates.

The long-term stability of a SERS substrate, in addition to repeatability and reproducibility, is a critical factor in determining whether the platform can deliver reliable performance for practical sensing applications. To assess the stability of the Fe_3_O_4_@C@TiO_2_ substrate, a time-dependent experiment was conducted, in which SERS signals were recorded after storing the substrates for different durations. The storage conditions were chosen to reflect realistic analytical scenarios – sealed container kept under ambient laboratory temperature and pressure. Substrates were retrieved after 3, 7, 15, 25, 35, and 45 days, and SERS spectra of 0.1 mM MB were collected at randomly selected spots at each time point. As shown in Fig. 7e, the SERS spectra exhibit excellent stability over time, with both the characteristic Raman bands of MB and their corresponding intensities well preserved. The spectra acquired at 3, 7, 15, 25, and 35 days show negligible variations, with the signal deviation remaining below 5%. Even after 45 days of storage, the Fe_3_O_4_@C@TiO_2_ substrate still delivers strong, clearly resolved MB signals, with peak intensities nearly unchanged. This outstanding stability can be attributed to the fully semiconductor-based composition of the Fe_3_O_4_@C@TiO_2_ substrate, which undergoes minimal chemical or structural degradation over time. Such temporal robustness offers a substantial advantage for long-term SERS applications, allowing substrates to be stored under simple conditions while preserving their sensing performance. This not only enhances analytical convenience and reliability but also greatly extends the operational lifetime of the substrates, thereby reducing fabrication, storage, and overall analytical costs. Taken together – with RSD values below 5% for repeatability and reproducibility, combined with the excellent long-term signal stability – the Fe_3_O_4_@C@TiO_2_ semiconductor substrate demonstrates exceptional reliability. This level of robustness is particularly noteworthy given that noble-metal-based SERS substrates often suffer from aging, surface oxidation, and unstable hot-spot distributions. The Fe_3_O_4_@C@TiO_2_ system thus represents a highly promising candidate for real-world SERS applications where consistency, durability, and practical usability are essential.

### 3.4. SERS sensing practicability of semiconductor Fe_3_O_4_@C@TiO_2_ nanostructures

**Fig. 8:**
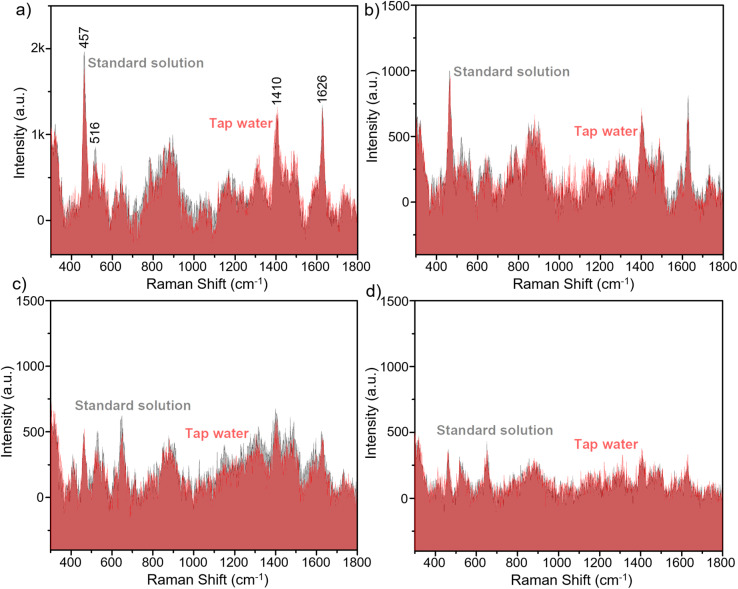
Comparison of the SERS signals of MB in real tap-water samples and standard solutions on the Fe_3_O_4_@C@TiO_2_ semiconductor substrate at concentrations of 5 × 10^−6^ M (a), 10^−6^ M (b), 5 × 10^−7^ M (c), and 10^−7^ M (d).

In practical applications, SERS substrates must not only deliver strong and stable signals but also sustain their performance under complex sample matrices and potential interferents. Having established the high sensitivity and reliable signal reproducibility of the semiconductor Fe_3_O_4_@C@TiO_2_ substrates, it is therefore crucial to evaluate their practicability in real-world sensing conditions. To this end, a demonstration experiment was conducted to detect the organic dye MB in tap water, aiming toward applications in monitoring contamination in household water supplies. Based on the analytical calibration established in Section 3.2, where a linear correlation (*R*^2^ = 0.98) between MB concentration and SERS intensity was obtained over the range of 5 × 10^−5^ M to 1 × 10^−7^ M, a series of tap-water samples spiked with MB at concentrations within this linear range was prepared and directly drop-cast onto the Fe_3_O_4_@C@TiO_2_ SERS substrates. Substrate preparation and spectral acquisition followed the procedures described in Section 2.3. The resulting SERS spectra, shown in Fig. 8 and compared with those of standard MB solutions, reveal clear and well-resolved MB Raman features across all tested concentrations. Importantly, the characteristic bands appear without significant spectral shifts relative to the standard solutions, indicating minimal matrix interference from tap water. Notably, even at a very low concentration of 10^−7^ M (Fig. 8d), the diagnostic MB Raman peaks remain clearly identifiable above the background noise, demonstrating excellent sensing capability. Furthermore, the peak intensities recorded from tap-water samples closely match those obtained from the corresponding standard solutions, underscoring the robustness of the Fe_3_O_4_@C@TiO_2_ substrate in real-word environments. For quantitative evaluation, detected MB concentrations were calculated using the linear regression equation *y* = 937 × *x* + 6669 established in Section 3.2, and the recovery values were determined by comparing detected concentrations with the spiked concentrations (Table 2). The recovery values fall within the range of 93% to 106%, demonstrating excellent accuracy and precision of the Fe_3_O_4_@C@TiO_2_ substrate for quantitative analysis in real matrices. These results collectively demonstrate that the semiconductor Fe_3_O_4_@C@TiO_2_ nanostructured substrate possesses outstanding practicability for real-world SERS applications, providing reliable and accurate detection performance.

**Table 2:** Practicability of semiconductor Fe_3_O_4_@C@TiO_2_ substrate for MB detection in tap water

Spiked MB concentration (M)	Detected MB concentration at 475 cm^−1^ (M)	Detected MB concentration at 1410 cm^−1^ (M)	Detected MB concentration at 1626 cm^−1^ (M)	Recovery (%)
5 × 10^−5^	4.92 × 10^−5^	4.95 × 10^−5^	4.87 × 10^−5^	98
1 × 10^−5^	9.81 × 10^−6^	8.92 × 10^−6^	9.20 × 10^−6^	93
5 × 10^−6^	4.90 × 10^−6^	5.28 × 10^−6^	5.13 × 10^−6^	102
1 × 10^−6^	1.02 × 10^−6^	1.21 × 10^−6^	8.86 × 10^−7^	103
5 × 10^−7^	4.98 × 10^−7^	4.87 × 10^−7^	4.91 × 10^−7^	98
1 × 10^−7^	9.95 × 10^−8^	1.07 × 10^−7^	1.11 × 10^−7^	106

## 4. Conclusions

In conclusion, we have developed a metal-free SERS substrate based on Fe_3_O_4_@C@TiO_2_ semiconductor heterostructures that delivers a sensitivity approaching that of noble-metal SERS substrates, together with excellent reliability and practical applicability. The engineered interfacial states within the heterostructure promote efficient semiconductor-molecule charge transfer, resulting in a pronounced amplification of the Raman response of methylene blue. Consequently, the substrate achieves an enhancement factor of 5.4 × 10^5^ and a detection limit of 76 nM – performance metrics that rival noble-metal systems and surpass most reported semiconductor-based SERS platforms. The material further exhibits outstanding stability and signal reproducibility, as evidenced by sustained SERS activity over prolonged storage and RSD values below 5%. Its capability for accurate MB detection in tap water, with recoveries of 93–106%, confirms its strong applicability in real analytical environments. Importantly, the Fe_3_O_4_@C@TiO_2_ heterostructure demonstrates robust SERS performance across different excitation energies, maintaining strong Raman responses under both low-energy (785 nm) and high-energy (532 nm) excitation conditions. This behavior highlights the effectiveness of interfacial electronic structure engineering in enabling efficient charge-transfer processes over a broad excitation window. Collectively, these results demonstrate that rational interface engineering can unlock metal-comparable SERS performance in semiconductor architectures, yielding a stable, scalable, and cost-effective sensing platform that advances SERS toward practical, on-site deployment.

## Author contributions

Q. D. Mai: conceptualization, methodology, investigation, formal analysis, writing – original draft, writing – review & editing; D. T. H. Trang: validation, investigation, data curation; N. T. Loan: validation, investigation; O. V. Hoang: validation, investigation; T. N. Bach: validation, investigation; N. Q. Hoa: validation, investigation; N. H. T. Tran: validation, writing – review & editing; A. T. Pham: methodology, writing – review & editing; A. T. Le: conceptualization, methodology, supervision, project administration, writing – review & editing.

## Conflicts of interest

The authors declare that they have no known competing financial interests or personal relationships that could have appeared to influence the work reported in this paper.

## Supplementary Material

RA-016-D6RA00307A-s001

## Data Availability

The data that support the findings of this study are available from the corresponding author upon reasonable request. Supplementary information (SI): calculations of the limit of detection (LOD), enhancement factor (EF), and relative standard deviation (RSD). See DOI: https://doi.org/10.1039/d6ra00307a.
