# Supplementary material for: Charge-transfer-activated SERS detection of methylene blue using an ultrastable, reliable, and highly sensitive semiconductor Fe3O4@C@TiO2 nano-platform
Source: RSC Adv. 2026 Apr 22;16(23):21152–67. doi: 10.1039/d6ra00307a (PMC13100877; doi:10.1039/d6ra00307a)
Supplement: RA-016-D6RA00307A-s001 [file RA-016-D6RA00307A-s001.pdf]

Supplementary Information (SI) for RSC Advances.
This journal is © The Royal Society of Chemistry 2026

Supporting Information for

Charge-Transfer-Activated SERS Detection of Methylene Blue Using an Ultrastable, Reliable, and Highly Sensitive Semiconductor Fe_3O_4@C@TiO_2 Nano-Platform

Quan-Doan Mai[a,*,1], Dang Thi Hang Trang[a,1], Ngo Thi Loan[a], Ong Van Hoang[b,a], Ta Ngoc Bach[c], Nguyen Quang Hoa[d], Nhu Hoa Thi Tran[e,f], Anh-Tuan Pham[g,a], Anh-Tuan Le[a,**]

[a]*Phenikaa University Nano Institute (PHENA), Phenikaa School of Engineering (PSE), Phenikaa University, Hanoi 12116, Vietnam*

[b]*University of Transport Technology, Trieu Khuc, Thanh Xuan District, Hanoi, Viet Nam*

[c]*Institute of Materials Science (IMS), Vietnam Academy of Science and Technology, 18 Hoang Quoc Viet, Hanoi 10000, Vietnam*

[d]*Faculty of Physics, VNU University of Science, Vietnam National University, Hanoi, Thanh Xuan, Hanoi, Vietnam*

[e]*Faculty of Materials Science and Technology, University of Science, Ho Chi Minh City, Vietnam*

[f]*Vietnam National University, Ho Chi Minh City, Vietnam*

[g]*Faculty of Biotechnology, Chemical and Environmental Engineering (BCEE), Phenikaa School of Engineering (PSE), Phenikaa University, Hanoi 12116, Vietnam*

Corresponding authors:

*doan.maiquan@phenikaa-uni.edu.vn (Q.D. Mai)

**tuan.leanh@phenikaa-uni.edu.vn (A.T. Le)

[1] Q.D. Mai and D.T.H. Trang contributed equally to this work

Calculation of limit of detection (LOD)

The calibration curve for the linear detection range was obtained as follows:

$$Y = A + B \times Log(X) \tag{1}$$

where A and B represent the intercept and slope of the regression equation derived from the logarithmic plot of SERS intensity (Y) versus analyte concentration (X).

The LOD is calculated using the following equation(1):

$$LOD = 10^{\left[\left(Y_{blank} + 3SD\right)/Y_{blank} - A\right]/B} \tag{2}$$

where Y_{blank} and SD denote the SERS signal and the standard deviation of the blank sample, respectively.

The standard deviation (SD) was determined using the well-known expression:

$$SD = \sqrt{\frac{1}{n-1} \times \sum_{i}^{n} (x_i - x_{average})^2} \tag{3}$$

where x_i is the value obtained from the i-th measurement, and $x_{average}$ represents the average signal of the blank sample acquired over n replicate measurements.

Calculation of enhancement factor (EF)

The EF value was determined using a well-established equation that has been widely adopted in previous studies(2, 3):

$$EF = \frac{I_{SERS}}{I_{Raman}} \times \frac{N_{bulk}}{N_{surface}} \tag{4}$$

where I_{SERS} and I_{Raman} denote the Raman intensities of the analyte measured with and without the SERS substrate, respectively. N_{bulk} represents the number of analyte molecules sampled in the conventional Raman measurement, whereas $N_{surface}$ corresponds to the number of molecules probed under SERS conditions.

N_{bulk} can be calculated following:

$$N_{bulk} = \frac{A_{laser} \times h \times \rho}{M} \times N_A$$

(5)

where A_{laser}, h, ρ and m represent the laser spot area, focal length, density of the solid analyte, and its molecular weight, respectively; and N_A is Avogadro's number.

$N_{surface}$ can be expressed as:

$$N_{surface} = \frac{C \times V}{A_{substrate}} \times N_A \times A_{laser} \tag{6}$$

where C, V, $A_{substrate}$ denote the analyte concentration, the drop-casted volume, and the substrate area, respectively; N_A is the Avogadro's number; and A_{laser} is the laser spot area.

Thus EF can be calculated as:

$$EF = \frac{I_{SERS}}{I_{Raman}} \times \frac{N_{bulk}}{N_{surface}} = \frac{h \times \rho \times A_{substrate}}{M \times C \times V} \tag{7}$$

In our case, I_{Raman} is Raman signal intensity without SERS substrate of methylene blue (MB), h = 2 µm, ρ_{MB} = 1.757 g/cm^3, M_{MB} = 320 g/mol, $A_{substrate}$ = 4 mm^2, V = 5 µL.

Calculation of relative standard deviation (RSD)

The RSD values for repeatability and reproducibility were determined using the standard formula:

$$\text{RSD} = \frac{SD \times 100}{x_{average}} \tag{8}$$

where SD, determined from Eq. (3), represents the standard deviation, while $x_{average}$ is the average SERS intensity derived from the repeated measurements.

References

1. Chen R, Shi H, Meng X, Su Y, Wang H, He Y. Dual-amplification strategy-based SERS chip for sensitive and reproducible detection of DNA methyltransferase activity in human serum. Analytical chemistry. 2019;91(5):3597-603.

2. Le Ru EC, Blackie E, Meyer M, Etchegoin PG. Surface enhanced Raman scattering enhancement factors: a comprehensive study. The Journal of Physical Chemistry C. 2007;111(37):13794-803.

3. Fu WL, Zhen SJ, Huang CZ. One-pot green synthesis of graphene oxide/gold nanocomposites as SERS substrates for malachite green detection. Analyst. 2013;138(10):3075-81.
